# Work Habit-Related Sleep Debt; Insights From Factor Identification Analysis of Actigraphy Data

**DOI:** 10.3389/fpubh.2021.630640

**Published:** 2021-03-10

**Authors:** Yuki Goto, Koichi Fujiwara, Yukiyoshi Sumi, Masahiro Matsuo, Manabu Kano, Hiroshi Kadotani

**Affiliations:** ^1^Department of Systems Science, Kyoto University, Kyoto, Japan; ^2^Department of Material Process Engineering, Nagoya University, Nagoya, Japan; ^3^Department of Psychiatry, Shiga University of Medical Science, Otsu, Japan; ^4^Department of Sleep and Behavioural Sciences, Shiga University of Medical Science, Otsu, Japan

**Keywords:** weekday sleep debt, actigraphy, machine learning, feature importance, support vector machine

## Abstract

The present study investigates the factors of “Weekday sleep debt (WSD)” by comparing activity data collected from persons with and without WSD. Since it has been reported that the amount of sleep debt as well the difference between the social clock and the biological clock is associated with WSD, specifying the factors of WSD other than chronotype may contribute to sleep debt prevention. We recruited 324 healthy male employees working at the same company and collected their 1-week wrist actigraphy data and answers to questionnaires. Because 106 participants were excluded due to measurement failure of the actigraphy data, the remaining 218 participants were included in the analysis. All participants were classified into WSD or non-WSD groups, in which persons had WDS if the difference between their weekend sleep duration and the mean weekday sleep duration was more than 120 min. We evaluated multiple measurements derived from the collected actigraphy data and trained a classifier that predicts the presence of WSD using these measurements. A support vector machine (SVM) was adopted as the classifier. In addition, to evaluate the contribution of each indicator to WSD, permutation feature importance was calculated based on the trained classifier. Our analysis results showed significant importance of the following three out of the tested 32 factors: (1) WSD was significantly related to persons with evening tendency. (2) Daily activity rhythms and sleep were less stable in the WSD group than in the non-WSD group. (3) A specific day of the week had the highest importance in our data, suggesting that work habit contributes to WSD. These findings indicate some WSD factors: evening chronotype, instability of the daily activity rhythm, and differences in work habits on the specific day of the week. Thus, it is necessary to evaluate the rhythms of diurnal activities as well as sleep conditions to identify the WSD factors. In particular, the diurnal activity rhythm influences WSD. It is suggested that proper management of activity rhythm may contribute to the prevention of sleep debt.

## Introduction

Sleep debt has deleterious effects on work or academic performance and also may impair various other psychological and physical functions such as memory, learning, metabolism, and immunity (1). However, a convenient method for quantitively evaluating sleep debt has not yet been established. According to the International Classification of Sleep Disorders, Third Edition (ICSD-3), getting enough sleep duration for at least 7 days before polysomnography (PSG) should be performed for the sake of sleep debt resolution (2); however, inventories to measure the degree of sleep debt have not been developed.

We focus on “weekday sleep debt (WSD),” which refers to taking longer sleep on the weekend to compensate for lack of sleep during the weekdays (3, 4). WSD is defined as the difference between the weekend and weekday sleep durations (5).

It has been well-known that chronotype affects weekday and weekend sleep timing and duration (6). One cause of WSD may be social jetlag, which is the discrepancy between biological and social clocks (7, 8). WSD may not occur when persons live following their born chronotype determined by genetic factors. However, it is difficult for most persons to live a daily social life by following their chronotype due to work or school. The factors of WSD other than chronotype should be specified to prevent sleep debt because the chronotype are reported to be influenced by genetic factors (5) as well as age, and personality (9).

It is assumed that persons with WSD may not get enough sleep during the weekdays and take longer sleeps on the weekend than during the weekdays to compensate for lack of weekday sleep. Actigraph devices have been widely used for long-term circadian activity measurement because they are lightweight, easy-to-wear, and non-invasive (10–12). The long-term activity measurement enables the evaluation of the discrepancy between biological and social rhythms clocks.

In this study, we aim to investigate the factors of WSD by using 1-week wrist actigraphy data. The actigraphy data and answers to questionnaires of persons with and without WSD were collected from 324 male employees at a Japanese wholesale company, and 218 participants were analyzed.

Before analysis, we validated the collected actigraphy data through comparison with sleep diaries recorded by the participants. In addition, the definition of WSD was examined with the chorotype of the participants determined by the Morningness-Eveningness Questionnaire (MEQ).

We trained a classifier that predicts the presence of WSD from the collected actigraphy data by utilizing a machine learning (ML) technique and calculated feature importance based on the trained classifier to specify the factors of WSD in addition to statistical analysis of the answers to questionnaires.

## Materials and Methods

### Measurements From Actigraphy Data

We calculated the following 40 parameters listed in Table 1 from the actigraphy data, whose details are described below.

- Sleep/Awake State: Wake−up time (WU), Sleep-onset time (SO), Mid−sleep (MS), and Sleep duration (SD)

**Table 1:** Measurements from actigraphy data.

**Explanatory variable**	**Abbreviations**
Wake-up time	WU_Tue_, WU_Wed_, WU_Thu_, WU_Fri_, WU_mean_, WU_std_
Sleep-onset time	SO_Mon_, SO_Tue_, SO_Wed_, SO_Thu_, SO_mean_, SO_std_
Mid-sleep	MS_Mon_, MS_Tue_, MS_Wed_, MS_Thu_, MS_mean_, MS_std_
Sleep duration	SD_Mon_, SD_Tue_, SD_Wed_, SD_Thu_, SD_mean_, SD_std_
Average activity during the most active 10-h period	M10_Tue_, M10_Wed_, M10_Thu_, M10_Fri_
Average activity during the least active 5-h period	L5_Tue_, L5_Wed_, L5_Thu_, L5_Fri_
Sleep regularity index	SRI
Sleep timing index	STI
Inter-daily stability	IS_act_, IS_light_
Intra-daily variability	IV_act_, IV_light_
Mid-sleep on free days	MSF
Social jetlag	SJL

The Cole-Kripke sleep/wake identification method was adopted to estimate wake-up and sleep-onset times from the actigraphy data (13). It has been reported that the agreement rate between the Cole-Kripke method and PSG was 88% (13, 14).

It is difficult to estimate sleep/awake timing using only the Cole-Kripke method because it uses 1-min activity counts data measured by means of actigraphy; therefore, we introduce a sleep/wake function *r*(*i*)

(1) $$\begin{array}{r} r\left(i\right)=\overset{i}{\underset{j=0}{\sum }}x_{j} \end{array}$$

where *x*_*i*_ is a sleep/wake state at the time *i* estimated by means of the Cole-Kripke method: *x*_*i*_ = +1 and *x*_*i*_ = −1 denote sleep and awake, respectively. *r*(*i*) becomes large when the sleep state dominates and becomes small when the wake state dominates. The extreme points of *r*(*i*) may be times that the sleep/awake states change. Thus, its maximum and minimum points can be defined as the wake-up time and the sleeping time. If there are multiple maximum and minimum points within a day, we adopt the latest one as the wake-up time or the sleeping time.

Using the sleep/wake function *r*(*i*), the following measurements, which represent the sleep habits, are estimated: wake-up time (WU), sleep-onset time (SO), mid-sleep (MS), and sleep duration (SD). WU, SO and MS are expressed as the minutes elapsed from midnight. Their means and standard deviations were used in addition to these values for each weekday.

- Sleep Rhythm: Sleep regularity index (SRI) and Sleep timing index (STI)

Sleep regularity index (SRI) (15) and sleep timing index (STI) (16) represent sleep rhythm. SRI is the agreement between sleep/wake states at any two-time points separated by 24 h. A large SRI means that the sleep rhythm is regular. SRI can be calculated based on the Cole-Kripke sleep/wake identification method. *N* and *p* are the numbers of measurement days and samples per day, respectively. *s*_*k,j*_ = 1 is satisfied if a person is determined to be in the sleep state at time *j* on the *k*th day, and *s*_*k,j*_ = 0 is satisfied if a person is determined to be in the awake state at time *j* on the *k*th day. SRI is defined as

(2) $$\begin{array}{r} \text{SRI }=\;-100+\;\frac{200}{p\left(N-1\right)}\overset{p}{\underset{j=1}{\sum }}\overset{N-1}{\underset{k=1}{\sum }}\delta_{s_{k,j},s_{k+1,j}} \end{array}$$

where δ_*i,j*_ = 1 if *i* = *j* and δ_*i,j*_ = 0 otherwise.

**Figure 1:**
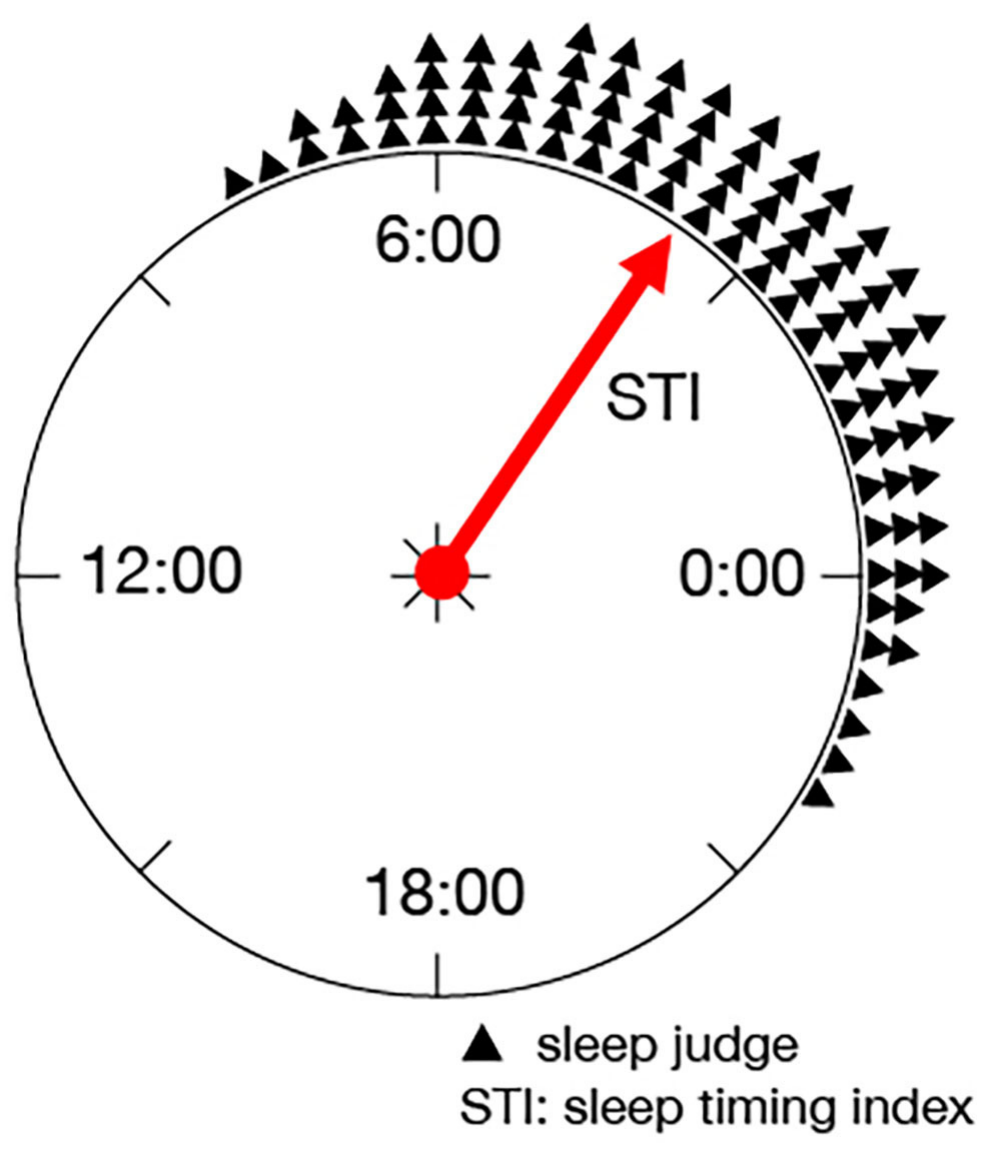
Schematic diagram of STI: STI expresses the mean sleep midpoint during the measurement days as the argument on a unit circle.

STI expresses the mean sleep midpoint during the measurement days. When the time from 0:00 to 24:00 are associated with the angles θ = [0, 2π], a time point *j* is expressed as θ_*j*_ = 2π*j*/*p*. Using this relationship, STI is defined as

(3) $$\begin{array}{r} \Theta \;=\;\arg\left(\overset{p}{\underset{j=1}{\sum }}\overset{N}{\underset{k=1}{\sum }}s_{k,j}e^{i\theta_{j}}\right) \end{array}$$

(4) $$\begin{array}{r} \text{STI }=\;\frac{60\times 24}{2\pi }\Theta \end{array}$$

where *i* is the imaginary unit, and Θ is the argument of the sum of $e^{i\theta_{j}}$ over the sleep states. Figure 1 shows a schematic diagram of STI, in which the triangles denote the times estimated as the sleep states, and $e^{i\theta_{j}}$ is the composition of the vectors directing these triangles. Thus, the time corresponding to the argument of $e^{i\theta_{j}}$ is STI.

- Activity Rhythm: Inter-daily stability (IS) and Intra-daily variability (IV)

Inter-daily stability (IS) and intra-daily variability (IV) are well-known parameters for activity rhythm evaluation based on the actigraphy data (17), which are defined as follows:

(5) $$\begin{array}{r} \text{IS }=\;\frac{M\sum_{j=1}^{p}{\left({\bar{A}}_{j}-\bar{A}\right)}^{2}}{p{\sum_{i=1}^{M}\left(A_{i}-\bar{A}\right)}^{2}} \end{array}$$

(6) $$\begin{array}{r} \text{IV }=\;\frac{M\sum_{i=2}^{M}{\left(A_{i}-A_{i-1}\right)}^{2}}{\left(M-1\right){\sum_{i=1}^{M}\left(A_{i}-\bar{A}\right)}^{2}} \end{array}$$

where *A*_*i*_ is the activity data at time *i*, ${\bar{A}}_{j}$ is the average of the activity data at time *j* over different days, and $\bar{A}$ is the average of all activity data. *p* is the number of activity data per day, and *M* is the total number of collected activity data. IS is the ratio of the variance of all the collected activity data to the variance of the average activity data at time *j* over different days. IS becomes large when the fluctuation of the diurnal activity data recorded at the same time of different days becomes large. IV is the ratio of the variance of the collected activity data to the sum of squares of the differences in activity data between a time point and the previous time point. IS and IV are measured for the synchronization of diurnal activity rhythm and the frequency of midday naps and nocturnal awakening, respectively (18).

IS and IV can be calculated from both the activity counts and illuminance in the actigraphy data, which are denoted as IS_act_, IS_light_, IV_act_, IV_light_, respectively.

- Rest–Activity Rhythm: Average activity during the most active 10-h period (M10) and Average activity during the least active 5-h period (L5)

Although IV represents intra-daily activity rhythm, M10 and L5 can be calculated from the actigraphy data to emphasize the rest-activity circadian rhythm. M10 and L5 are defined as the average activities during the most active 10-h period and during the least active 5-h period, respectively (17). M10 represents activity during the most active period within the day, which may be influenced by daytime napping. L5 represents movement activity during sleep.

- Biological Clock: Mid-sleep on free days (MSF) and Social jetlag (SJL)

Mid-sleep on free days (MSF) is a measurement of chronotype. Wake-up time on free days (WUF) and sleep-onset time on free days (SOF) are wake-up time and sleep onset time on the weekend, respectively. MSF is expressed as

(7) $$\begin{array}{r} \text{MSF }=\;\frac{\text{SOF}+\text{ WUF}}{2} \end{array}$$

Although sleep during the weekdays does not reflect the congenital biological clock much due to the effect of the social clock, weekend sleep significantly reflects the congenital biological clock. Chronotype is classified into three types based on MSF: morning type (MSF < 3:00), intermediate type (3:00 ≤ MSF ≤ 4:00), and evening type (MSF > 4:00) (19).

In addition, social jetlag (SJL) is the difference between the biological clock and the social clock, which is defined as

(8) $$\begin{array}{r} \text{SJL }=\text{ MSF}-\text{MSW} \end{array}$$

where MSW is the meantime of mid-sleep on the weekdays.

Although the Morningness Eveningness Questionnaire (MEQ) (20) or the Munich Chronotype Questionnaire (MCTQ) (21) is usually used for calculating MSF and SJL, we used the actigraphy data for calculating them instead of the questionnaires in this study.

### Measurements From Questionnaires

In addition to the measurements derived from the actigraphy data, we collected answers to the following, modified for the Japanese population.

- The Zung Self-rating Depression Scale (SDS) (22, 23): a 20-item quantitative measurement of symptoms of depression. The subjects rate each item regarding how they felt during the week preceding. Scores of ≤ 39, 40–49, and ≥ 50 on the SDS indicate no, mild, and moderate-to-severe depressive symptoms, respectively.
- The Epworth Sleepiness Scale (ESS) (24, 25): an eight-item questionnaire that is widely used for assessment of daytime sleepiness in adults. ESS is used to assess the severity of insomnia with a possible score range of 0–24 points. An ESS score of 11 points or higher indicates excessive daytime sleepiness.
- The Pittsburgh Sleep Quality Index (PSQI) (26, 27): a 19-item self-rated questionnaire assesses sleep quality. Nineteen individual items generate seven component scores: subjective sleep quality, sleep latency, sleep duration, habitual sleep efficiency, sleep disturbances, use of sleeping medication, and daytime dysfunction. The sum of scores for these seven components yields one global score. PSQI global score of > 5 indicates poor sleep quality.
- The Social Rhythm Metric (SRM) (28): a diary-like questionnaire that quantifies the extent to which a person's life is regular on a daily basis with respect to event timing. It gives a score based on the timing of five activities that are thought to constitute an individual's social rhythm (1: Get out of bed, 2: First contact with another person, 3: Start work, housework, or volunteer activities, 4: Have dinner, and 5: Go to bed), The SRM-score lies on a continuum between 0 and 7, with 0 representing lowest regularity and seven highest regularity.
- The 36-item Short-Form Health Survey (SF-36) (26, 29): a 36-item questionnaire that evaluates the health-related quality of life (QOL) from eight components: physical functioning, role limitations–physical, bodily pain, general health, vitality, social functioning, role limitations–emotional, and mental health. For each subscale, a score ranging from 0 (worst) to 100 (best) is calculated and standardized to have a mean of 50 and a standard deviation of 10.
- Morningness-Eveningness Questionnaire (MEQ) (20): a 19-item self-rated questionnaire, each having four or five response options. The last question, item 19, asks whether a participant estimates oneself as definitely a morning type, rather more a morning type than an evening type, rather more an evening type than a morning type, or definitely an evening type. The sum gives a score ranging from 16 to 86. Scores of 70–86, 59–69, 42–58, 31–41, and 16–30 are classified as definitely morning type, moderately morning type, neither type, moderately evening type, and definitely evening type, respectively.

### Definition of Weekday Sleep Debt

Sleep duration (SD) of each day can be calculated using the sleep/wake function *r*(*i*). By using the means of SD on the weekend (SD on a free day; SDF) (min), and SD on the weekday (SDW) (min), sleep rebound on the weekend (SRW) (min) (30) is defined as

(9) $$\begin{array}{r} \text{SRW }=\text{ SDF}-\text{SDW} \end{array}$$

A person may have chronic sleep deprivation during the weekdays when SRW ≥ 120 min (31). Based on this criterion, we judge that a person has WSD when SRW ≥ 120 min.

### Machine Learning-based Factor Identification

In this research, we adopt machine learning (ML) technologies to find the factors of WSD. A classifier that predicts whether a person has WSD is trained from the measurements described above or not, and feature importance is calculated based on the trained classifier. Feature importance is a well-known method for identifying which input features in a classifier contribute to output (32).

Although various classifier training methods have been used in ML, we adopted a support vector machine (SVM) in this work. SVM is a classical nonlinear classification technique that was originally developed for classifying data into two classes (33). SVM has been widely used for various applications such as spam mail filtering, bioinformatics, and object recognition (34–36). Thus, SVM is a reliable ML technique.

We used a permutation feature importance method to calculate feature importance, which is defined as the decrease in prediction performance when a single input feature is randomly shuffled (37, 38). *E* is the prediction performance of the trained classifier when the original feature set is input to the classifier, and *E*_*j*_ is the prediction performance of the classifier when a feature *x*_*j*_ is exchanged randomly into another feature. The feature importance of *x*_*j*_ is calculated as

(10) $$\begin{array}{r} \text{Pimp}\left(x_{j}\right)\;=\;E-\;E_{j} \end{array}$$

We can judge that the feature *x*_*j*_ contributes to a prediction when *Pimp*(*x*_*j*_) is large.

### Data Description

A cross-sectional survey was conducted in 324 employees at a drug wholesale company in Osaka, Japan, from January 26, 2004, to December 19, 2005.

All of the participants were male. It becomes difficult to focus on the relationship between work habits and activity rhythm when female employees are included in the survey. There are more factors affecting sleep and rhythm in females than in males, such as having small children or not, menstrual cycle, unmarried or married (39, 40). However, some studies have already reported that there were no significant differences in daytime activities between males and females (41, 42). Thus, it is expected that our analysis is applicable to females as well as males.

The workdays were from Monday to Friday, and the holidays were Saturday and Sunday, which are typical for Japanese companies and government offices. We used a wrist-type actigraph device (Actiwatch AW-Light, Mini-Mitter) for data recording, which measures the activity counts and illuminance within every one-minute bin. Illuminance is recorded by a photodiode equipped in the actigraph device. Its measurement range is 0.1 to 150,000 Lux with 0.1 Lux resolution, which is suitable for both interior and exterior illuminance measurement. The actigraph device worked for 2–3 months per one battery. Thus, data missing did not occur during data collection. In addition, we collected answers to the questionnaires and sleep diaries during the survey. The protocol of this study was approved by the Ethics Committee of the Shiga University of Medical Science (R2020-026).

Since all participants wore the actigraph device from around 5:00 pm on a Monday to around 8:00 am on the next Monday, the Monday (the first day) actigraphy data were not recorded in their entirety. The Monday data were excluded from the analysis except for wake-up and sleep-onset time estimation. On the other hand, sleep-onset time, mid-sleep, and sleep duration of Friday were not able to be calculated because sleep in the Friday night sleep is regarded as a weekend.

One hundred and six participants who removed the actigraph device for more than 30 min during the survey term were excluded from the analysis so that 218 participants were included in the analysis. The characteristics of the subjects are illustrated in Table 2, and a detailed description of the data used in this study is available in Kadotani et al. (3), Nakayama-Ashida et al. (43), and Gerstner et al. (44).

**Table 2:** Characteristics of subjects.

Age	43.8 ± 8.4
BMI, kg/m^2^	23.7 ± 3.1
Current smoker	175
Habitual snorer, everyday or often	156
Habitual drinker, almost everyday	177
Alcohol consumption, g·d^−1^·kg^−1^	0.5 ± 0.5
Hypertension	50
Daytime sleepiness, ESS > 10	86
ESS score	8.1 ± 4.3
Total sleep time, h	5.9 ± 0.9
Sleep latency, min	10.8 ± 15.7
Sleeping pill use, yes	8

Our dataset consisted of 1-week actigraph data, which seems to be rather short for analysis. However, Tienoven et al. calculated SRM of healthy persons from their 1-week activity data (45). Fonseca et al. evaluated RM of stroke patients and healthy persons based on 1-week activity data (46). In addition, the sleep extension term required before performing PSG is also 1 week according to ICSD-3 (2). These indicate that 1-week activity data can be used for sleep rhythm evaluation.

**Figure 2:**
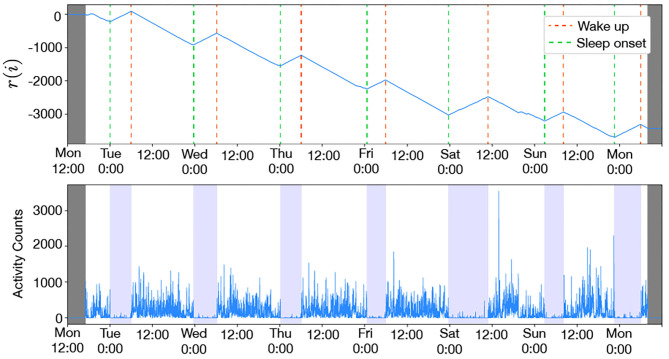
Example of wake-up and sleep-onset times estimated by activity-counts (male, 41 years old, MSF = 3:32): the colored bands in the bottom figure denote the duration between the estimated wake-up and sleep-onset. The sleep/wake function *r*(*i*) decreased during daytime and increased during the night, and the awake and sleep states repeated in the 24-h cycle. Since the awake state is longer than the sleep state, *r*(*i*) had a downward trend with the up and down periodic cycle.

**Figure 3:**
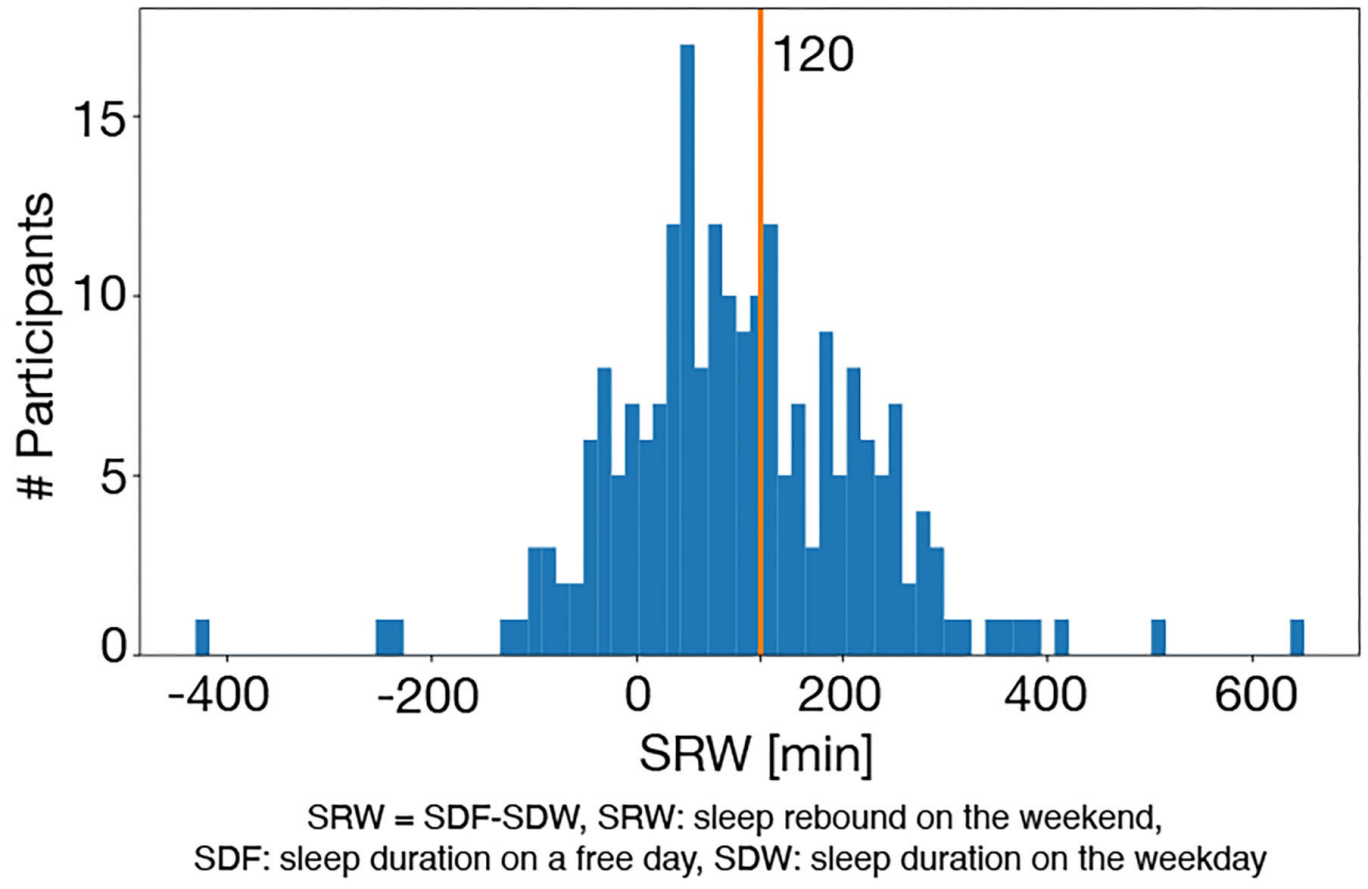
Distribution of SRW: the red vertical line denotes the cut-off value of WSD (120 min), and the right and left sides of this line are the WSD and the non-WSD groups.

An example of sleep/awake state estimation is shown in Figure 2. All participants were classified into the WSD group or the non-WSD group based on the SRW calculated from the actigraphy data. The numbers of persons with and without WSD were 89 and 129, respectively. Figure 3 shows the distribution of SRW in this data.

### Analysis Procedure

Before analysis, we validated the accuracy of the actigraphy data through a comparison between sleep durations derived from the actigraphy and the sleep diaries recorded by participants themselves. In addition, the answers of MEQ item 19, which asks self-awareness of chronotype were compared to verify whether the definition of WSD adopted in this study was supported by self-awareness of chronotype.

To specify the factors of WSD based on the feature importance, we needed to construct a classifier that predicts the presence of WSD. In general, the numbers of positive and negative examples for training should be balanced in binary classification problems to construct a good classifier (47). We randomly discarded non-WSD data so that the numbers of persons with WSD and non-WSD became even by means of random under-sampling because the number of persons without WSD was larger than that of persons with WSD.

As the input features of the WSD classifier, we used 40 features listed in Table 1, derived from the actigraphy data. The Gaussian kernel with a parameter γ was adopted in SVM, of which the parameter γ was tuned by means of 10-fold cross-validation. The WSD classifier training and permutation feature importance computation were repeated 100 times by changing the training and the test samples at random, in which the ratio of the number of training samples to that of test samples was fixed at 7:3. Finally, the mean of the computed permutation feature importance was calculated. In this study, we focus on features occupying the top 20% of the sum of importance, which does not necessarily mean only these factors are important (or statistically significant). We used the top 20% just for limiting the number of factors to discuss in detail, in which the cut-off value can be varied.

In addition, we examined whether the presence of WSD may relate to occupational categories or not. In this survey, employees were classified into the following categories: clerical, managers, professional and technical sales, service, transportation and communication, manufacturing, and others (43).

We compared each of the 40 input features and each of the collected answers to the questionnaires between the WSD and non-WSD groups by means of a statistical test.

### Statistical Analysis

We used the *t*-test for comparison between the WSD and non-WSD groups. The significance level was set to *p* < 0.05. To consider the multiple comparisons of the total of 40-measurements from the actigraphy data and 14-measurements from the questionnaires, the Bonferroni correction was adopted. That is, the significance levels were corrected as *p* < 0.05/40 = 0.00125 and *p* < 0.05/13 = 0.0038. In addition, we used the χ^2^ test with significance *p* < 0.05 for examining the effect of occupational categories on WSD and MEQ item 19.

Computation in this study was performed in Python 3.6.4 with SciPy 1.3.0, NumPy 1.16.2, and scikit-learn 0.20.2.

## Results

**Figure 4:**
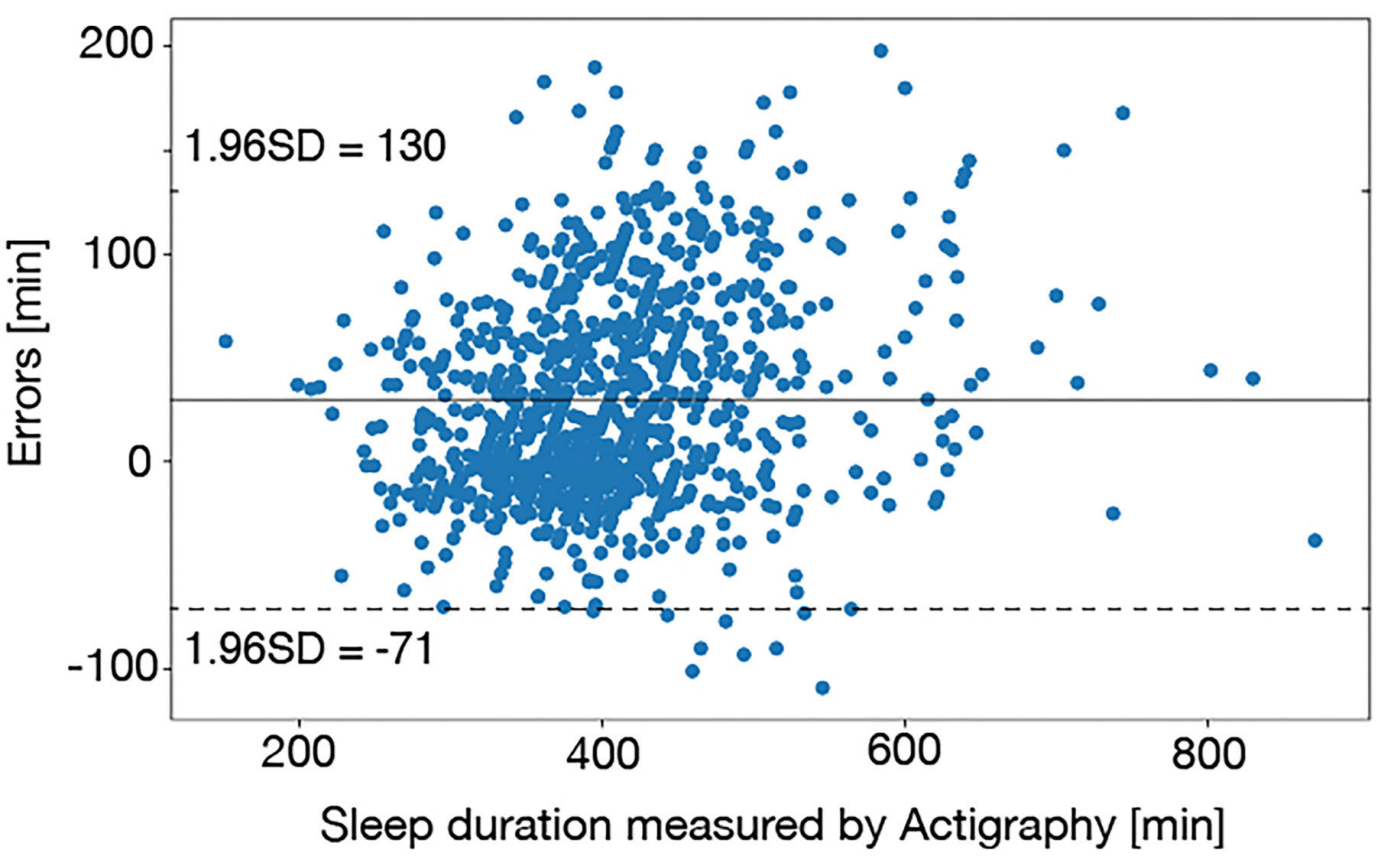
The Bland-Altman plot between sleep durations derived from the actigraphy and the sleep diaries. Most of the sleep duration errors were scattered within 1–1.5 h, which was appropriate accuracy.

First, we checked the validity of the actigraphy data. Figure 4 is the Bland-Altman plot between sleep durations derived from the actigraphy and the sleep diaries, which shows most of the sleep duration errors were scattered within −71 to 130 min. Thurman et al. (48) reported that there were errors of >1.5 h between sleep durations derived from the actigraphy and the sleep diaries in 79% of healthy adults. Short et al. (49) confirmed that the average errors between sleep durations derived from the actigraphy and the sleep diaries were 87 min in healthy adolescents. According to these studies, the sleep duration errors observed in this study was appropriate.

The participants distribution of MEQ item 19 is shown in Table 3, and a significant difference was confirmed in MEQ item 19 through the χ^2^ test (*p* = 0.12 × 10^−7^). On the other hand, there was no significant difference in the total score of MEQ (*p* = 0.12). Thus, the definition of WSD adopted in this study was associated with self-awareness of chronotype.

**Table 3:** The participants distribution in item 19 of MEQ.

	**Definitely a morning type**	**Rather more a morning type than an evening type**	**Rather more an evening type than a morning type**	**Definitely an evening type**
WSD	13	31	28	17
non-WSD	11	57	35	26

We compared the answers to the questionnaires between the WSD and non-WSD groups using the *t*-test with the Bonferroni correction. There were no questionnaires that had a significant difference between the WSD and non-WSD groups, as shown in Table 4.

**Table 4:** Comparison of questionnaires (significance level with the Bonferroni correction: *p* < 0.0038).

		**WSD**	**non-WSD**	***p*-value**
PSQI		4.86 ± 1.97	4.60 ± 1.84	0.34
ESS		8.28 ± 3.99	7.56 ± 4.29	0.23
SDS		36.6 ± 5.72	36.3 ± 6.34	0.75
SRM		4.63 ± 0.860	4.97 ± 0.874	0.0056
MEQ		53.7 ± 5.38	54.6 ± 5.45	0.12
SD-36	PF	53.6 ± 8.32	53.6 ± 5.11	0.93
	RP	52.6 ± 7.89	52.2 ± 6.88	0.72
	BP	50.4 ± 9.07	51.6 ± 9.44	0.36
	GH	52.1 ± 8.69	49.7 ± 9.43	0.063
	VT	49.7 ± 8.90	49.9 ± 8.00	0.88
	SF	52.7 ± 7.61	52.8 ± 7.37	0.98
	RE	51.5 ± 8.50	51.7 ± 7.21	0.81
	MH	49.8 ± 9.86	50.8 ± 8.32	0.44

**Figure 5:**
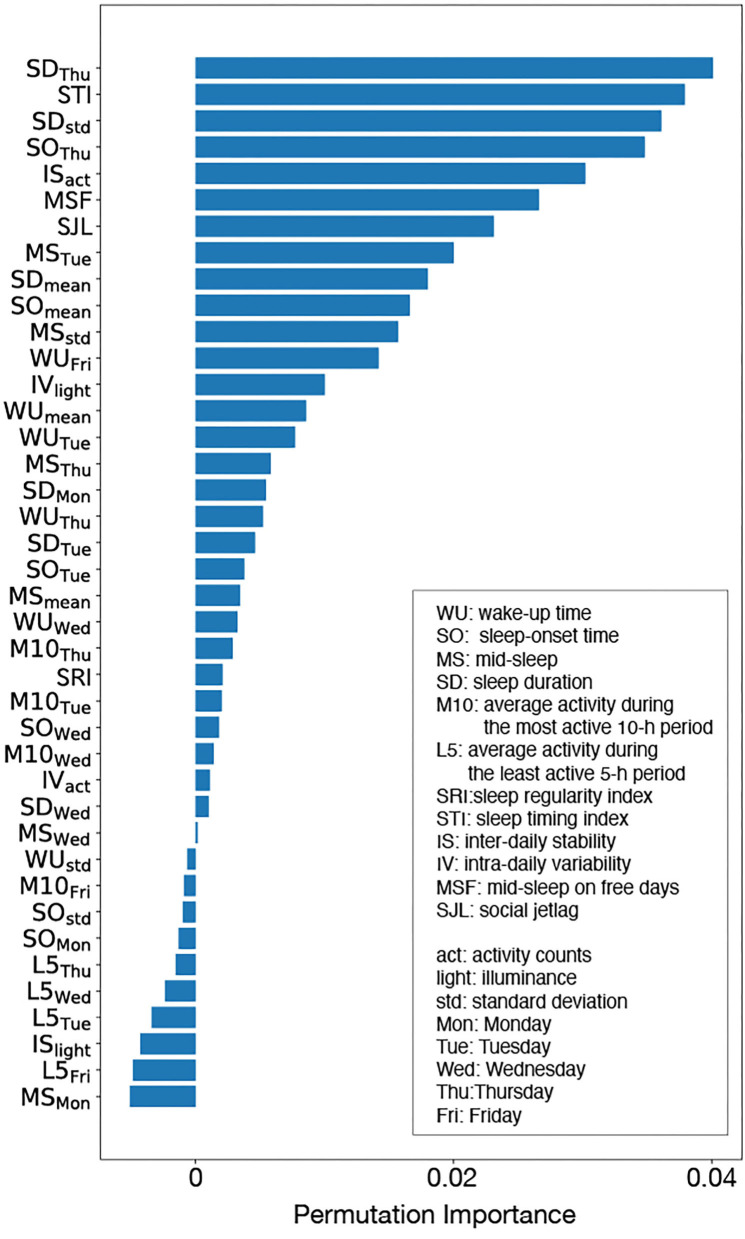
Permutation importance: although there were some features with negative importance, such features did not contribute to WSD prediction. Sleep duration on Tuesday (SD_Thu_), sleep timing index (STI), the standard deviation of sleep duration (SD_std_), sleep onset on Tuesday (SO_Thu_), inter-daily stability calculated by the activity counts (IS_act_ ), mid-sleep on free days (MSF), and social jetlag (SJL) were judged as the important features for WSD.

The calculated mean of the permutation feature importance is shown in Figure 5, which shows that the estimated WSD factors are as follows: sleep duration on Thursday (SD_Thu_), sleep timing index (STI), the standard deviation of sleep duration (SD_std_), sleep onset on Thursday (SO_Thu_), inter-daily stability calculated by the activity counts (IS_act_), mid-sleep on free days (MSF), and social jetlag (SJL).

Table 5 shows the comparison result of input features between the WSD and non-WSD groups by the *t*-test with the Bonferroni correction, in which features displayed by bold fonts have a significant difference between the two groups. In addition, the last column in Table 5 indicates the estimated WSD factors by the permutation feature importance. According to the *t*-test, MSF and SJL were significantly different, as well as some features about the weekday sleep condition, including SD_Thu_ and SO_Thu_. The results of the *t*-test almost agreed with those of the permutation importance. Thus, these results indicate that sleep on Thursday, activity rhythm and biological clock might contribute to WSD.

**Table 5:** Comparison of measurements between the two groups (significance level with the Bonferroni correction: *p* < 0.00125).

	**WSD**	**non-WSD**	***p*-value**	**Importance**
WU_Tue_	334 ± 49.1	348 ± 43.2	0.031	
WU_Wed_	335 ± 48.4	346 ± 61.0	0.15	
WU_Thu_	340 ± 55.8	349 ± 49.0	0.22	
WU_Fri_	342 ± 39.6	348 ± 48.1	0.30	
WU_mean_	338 ± 37.0	348 ± 41.9	0.065	
WU_std_	19.8 ± 24.7	18.3 ± 22.0	0.66	
SO_Mon_	1,446 ± 71.4	1,422 ± 62.6	0.012	
SO_Tue_	1,442 ± 81.5	1,420 ± 70.6	0.040	
SO_Wed_	1,419 ± 83.2	1,403 ± 76.4	0.17	
**SO**_**Thu**_	**1,429** **±** **71.2**	**1,395** **±** **71.4**	**0.00061**	*
SO_mean_	1,434 ± 58.8	1,410 ± 52.9	0.0025	
SO_std_	44.0 ± 25.6	41.1 ± 24.6	0.41	
MS_Mon_	170 ± 46.5	165 ± 42.3	0.43	
MS_Tue_	169 ± 43.8	163 ± 53.9	0.39	
MS_Wed_	159 ± 52.8	156 ± 47.9	0.65	
MS_Thu_	166 ± 43.7	152 ± 48.7	0.027	
MS_mean_	166 ± 36.7	159 ± 39.5	0.18	
MS_std_	24.3 ± 16.6	23.9 ± 15.3	0.84	
**SD**_**Mon**_	**328** **±** **79.7**	**366** **±** **66.6**	**0.00031**	
SD_Tue_	333 ± 101.5	366 ± 76.0	0.011	
SD_Wed_	361 ± 94.4	386 ± 85.5	0.053	
**SD**_**Thu**_	**352** **±** **75.0**	**393** **±** **73.1**	**0.00011**	*
**SD**_**mean**_	**344** **±** **65.3**	**378** **±** **53.5**	**0.000082**	
SD_std_	52.6 ± 30.9	45.3 ± 30.8	0.093	*
M10_Tue_	22,633 ± 6,991	21,362 ± 7,114	0.19	
M10_Wed_	22,019 ± 6,524	21,320 ± 7,200	0.58	
M10_Thu_	22,490 ± 6,784	21,134 ± 6,810	0.47	
M10_Fri_	23,555 ± 7,677	22,109 ± 7,406	0.22	
L5_Tue_	900 ± 655	953 ± 711	0.15	
L5_Wed_	741 ± 573	860 ± 765	0.36	
L5_Thu_	742 ± 654	830 ± 705	0.17	
L5_Fri_	796 ± 660	890 ± 851	0.38	
SRI	69.8 ± 7.51	70.2 ± 7.72	0.74	
STI	151 ± 39.9	147 ± 40.9	0.53	*
IS_act_	0.468 ± 0.0638	0.491 ± 0.072	0.015	*
IV_act_	0.591 ± 0.0943	0.573 ± 0.0928	0.16	
IS_light_	0.343 ± 0.0684	0.348 ± 0.0724	0.65	
IV_light_	0.725 ± 0.239	0.693 ± 0.233	0.33	
**MSF**	**255** **±** **108.3**	**210** **±** **78.2**	**0.0010**	*
**SJL**	**89** **±** **105.4**	**51** **±** **69.1**	**0.0034**	*

* *The estimated WSD factors by the permutation feature importance. Bold features indicate that have a significant difference between the two groups*.

**Figure 6:**
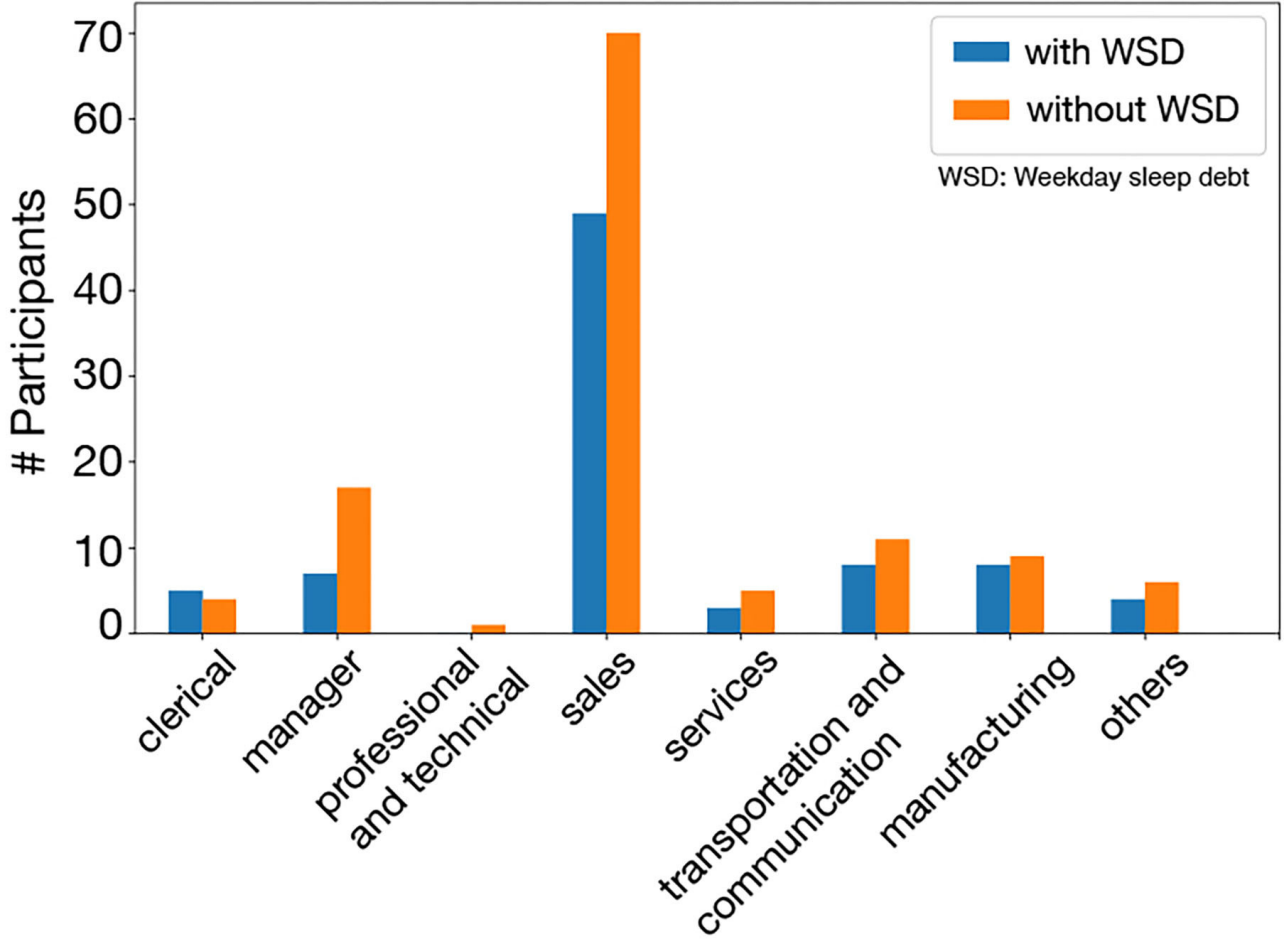
WSD and non-WSD groups in occupational categories: in all categories, there was not much difference between the WSD and the non-WSD groups.

Figure 6 shows the numbers of employees with/without WSD in each occupational category. The additional χ^2^ test examining the effect of occupational categories on WSD showed that the occupational category was not associated with the presence of WSD in this dataset (*p* = 0.87).

**Figure 7:**
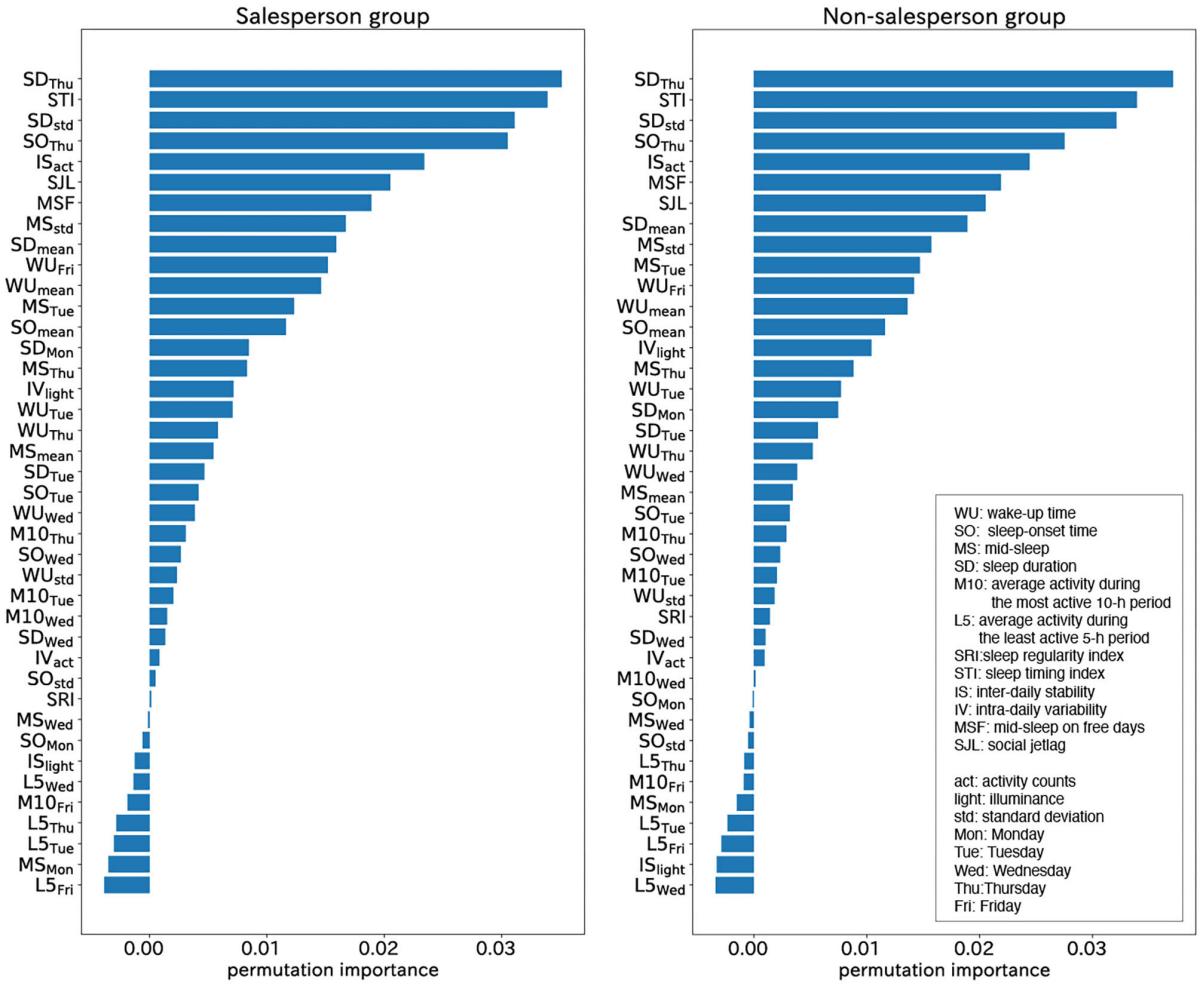
Permutation importance calculated from salesperson group (left) and non- salesperson group (right): the top five WSD factors were the same. The factors of WSD were not related to specific occupational categories.

We constructed additional two classifiers using the actigraphy data collected from two groups: a salesperson group (*N* = 119) and a non-salesperson group (*N* = 205). The calculated means of the permutation feature importance derived from each classifier were shown in Figure 7. The top five WSD factors were the same, which shows that the factors of WSD were not related to specific occupational categories.

## Discussion

### Chronotype and WSD

There was a significant difference in MEQ item 19 between WSD and non-WSD groups (*p* = 0.12 × 10^−7^) although a significant difference in the total score of MEQ was not confirmed (*p* = 0.12).

Turco et al. (50) assessed the reliability of MEQ item 19 in comparison with the total score of MEQ, the time of subjective sleepiness, and real-life sleep timing variables. They found that significant differences in sleep-wake timing between the answers of MEQ item 19. In addition, such differences were still observed when sleep-wake habits were analyzed separately on work and free days. In addition, Arrona-Palacios and Díaz-Morales (51) confirmed Turco's study in the Mexican and Spanish populations. These findings suggest that MEQ item 19 is solely effective for subjective chronotype evaluation as well as objective chronotype evaluation.

Thus, it is concluded that the definition of WSD supported by MEQ item 19 was appropriate from the viewpoint of chronotype.

### Day-to-Day Variance

The weekday sleep conditions were different between the WSD and non-WSD groups, according to Table 2. Sleep duration during the weekdays of the WSD group was significantly shorter than that of the non-WSD group except for Wednesday (*p* < 0.05), which may be plausible from the viewpoint of the assumption that WSD compensates for sleep deprivation during the weekdays. SD_Thu_ and SO_Thu_ were important features for WSD based on the analysis result of permutation importance analysis, which indicated that Thursday was associated with WSD.

### Morning Evening Tendency

There were significant differences in MSF and SJL, the biological clock features (*p* < 0.05). MSF and SJL of the WSD group were later than those of the non-WSD group. That is, persons with WSD have eveningness tendencies. In addition, MSF and SJL were also important to WSD according to the permutation importance analysis. Since all of the participants worked at the same company, they might live and work following the same social clock. Thus, this result indicated that disagreement between the social clock and the individual biological clock might contribute to WSD, which is consistent with the previous study (5).

### Activity Levels Variance

The inter-daily stability by the activity counts (IS_act_) evaluates the synchronization of diurnal activity rhythm, and it was also an important feature for WSD according to the permutation importance analysis, although the significant difference of IS_act_ between the WSD and the non-WSD groups was not confirmed. Figure 5 shows the 24-h activity counts during the weekdays of two participants with and without WSD. Inter-daily synchronization of activity rhythm, including sleep conditions, might affect WSD.

On the other hand, intra-daily variability based on the activity counts (IV_act_) was not significantly different between the WSD and non-WSD groups, and its feature importance was low. Since IV_act_ denotes the frequency of midday naps and nocturnal awakening, its low importance suggests that sleep disorders, such as narcolepsy or sleep apnea, might not contribute to WSD. In addition, IS and IV by illuminance (IS_light_ and IV_light_) were not significantly different and of low importance, implying that illuminance might not affect WSD.

**Figure 8:**
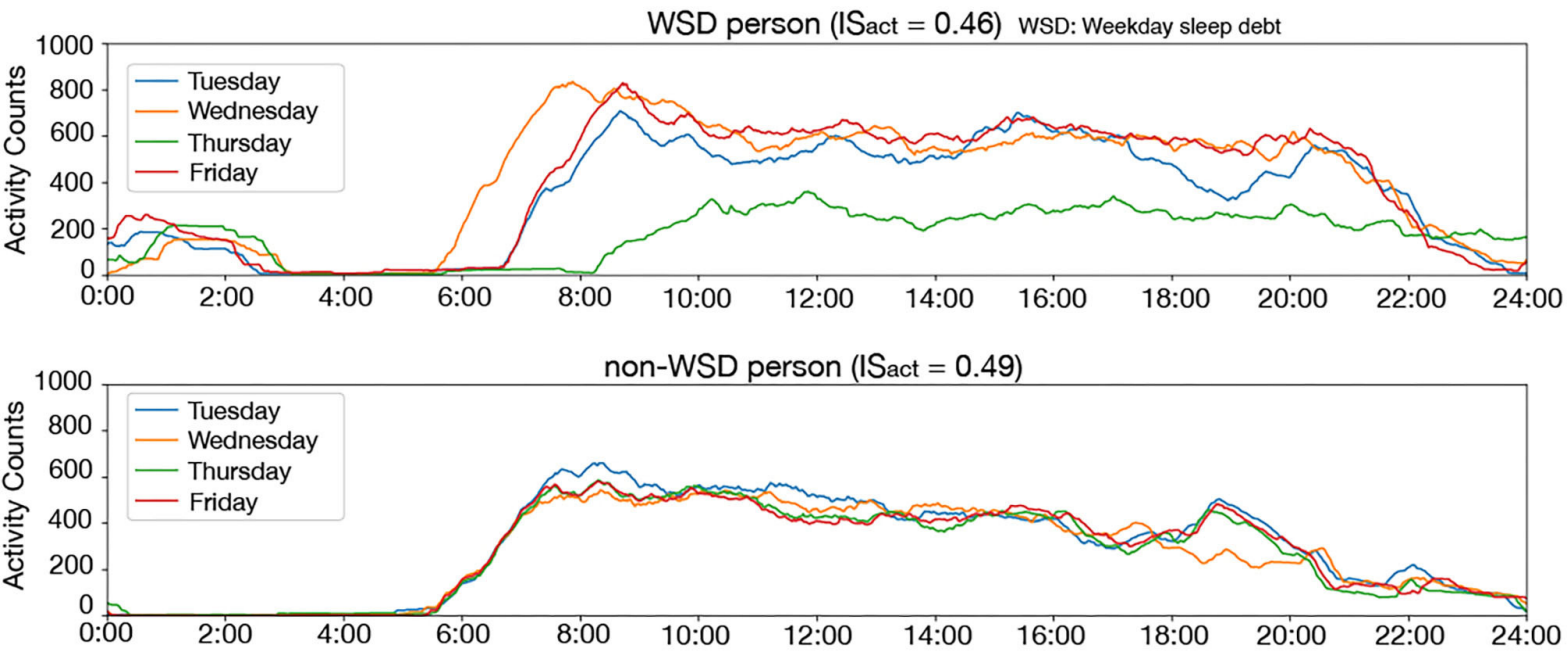
Twenty four-hours activity counts on the weekday of a person with WSD **(top)** and without WSD **(bottom)**: the activity counts are smoothed by using the 60-min moving average for clear display. This figure clearly illustrates that the activity counts on Thursday were clearly different from other days in a person with WSD.

It was of note that Thursday was more important for WSD presence than other weekdays. This may be related to the participants' working conditions. All participants worked at the same drug wholesale company, which provides various medical supplies to hospitals and clinics. Since private clinics in Japan tend to close on Thursday evening (52), work habits on Thursdays are different from other workdays at the drug wholesale company. For example, employees might tend to do desk work rather than outside work or to go home earlier than other workdays. Such differences in work schedules might affect their social clock and disturb the synchronization of diurnal activity rhythm, which is consistent with the causes of WSD (5). Figure 8 clearly illustrates that the activity of a participant with WSD on Thursday was significantly different from those of other days while the activity of a participant without WSD synchronized even on Thursday. This difference might indicate increased desk time and reduced outside work on Thursday.

Thus, it is reasonable that Thursday was an important factor of WSD at the company. This analysis indicates that the work style differences on a specific day of the week may cause WSD.

### Occupational Category

In this survey, participants were classified into a salesperson group and a non-salesperson group, and the top five permutation feature importance derived from each group was the same, as shown in Figure 6. This result indicated that the factors of WSD were not related to specific occupational categories, which also suggests that our analysis may be applicable to data collected from other companies.

The limitations of this study include the collected data; all of the participants were male and Japanese. Hence, we could consider neither gender nor racial differences in this study. In addition, the participants worked at the same company; it was difficult to investigate the effects of other days of the week on WDS since we did not compare our data with persons working at companies that have different work habits on a specific day of the week. Accordingly, we need to collect data from employees working in various types of industries to confirm our results. In addition, we were not able to analyze the Monday activity data due to the constraint of the data collection, which might affect the analysis results.

## Conclusion

In this study, we collected actigraphy data from 324 healthy male employees at a drug wholesaler and calculated permutation feature importance based on SVM to identify the factors of WSD. We compared the answers to questionnaires between the WSD and the non-WSD groups.

Our analysis results indicated that sleep duration during the weekdays and the individual biological clock might affect WSD, which is consistent with previous studies. In addition, we demonstrated a new finding that turbulence of diurnal activity rhythm synchronization as well as nocturnal sleep rhythm, even for 1 day, is associated with WSD, which is a new finding of this work.

In the future, we will develop a quantitative evaluation methodology for sleep debt based on this study.

## Data Availability Statement

The data analyzed in this study is subject to the following licenses/restrictions: The actigraphy data will be made available by the corresponding author to colleagues who propose a reasonable scientific request after approval by the institutional review board of the SUMS Hospital. Requests to access these datasets should be directed to Hiroshi Kadotani, kadotanisleep@gmail.com.

## Ethics Statement

The studies involving human participants were reviewed and approved by the Ethics Committee of the Shiga University of Medical Science. The patients/participants provided their written informed consent to participate in this study.

## Author Contributions

YG and KF analyzed the clinical data and composed the manuscript. YS, MM, and HK collected clinical data. MK and HK checked the analysis result and the manuscript. All authors contributed to the article and approved the submitted version.

## Conflict of Interest

KF is with Quadlytics Inc. as well as Nagoya University. MK is with Quadlytics Inc. as well as Kyoto University. HK's laboratory, the Department of Sleep and Behavioral Sciences is an endowment department, supported with unrestricted grants from Fukuda Lifetech Co., Ltd., Fukuda Life Tech Keiji Co., Ltd., Tanaka Sleep Clinic, Akita Sleep Clinic, and Ai Ai Care Co., Ltd. The remaining authors declare that the research was conducted in the absence of any commercial or financial relationship that could be construed as a potential conflict of interest.
